# Evaluating the use of 3'-(*p*-Aminophenyl) fluorescein for determining the formation of highly reactive oxygen species in particle suspensions

**DOI:** 10.1186/1467-4866-10-8

**Published:** 2009-08-11

**Authors:** Corey A Cohn, Christopher E Pedigo, Shavonne N Hylton, Sanford R Simon, Martin AA Schoonen

**Affiliations:** 1 Center for Environmental Molecular Science, Stony Brook University, Stony Brook, NY 11794-2100, USA; 2 Department of Geosciences, Stony Brook University, Stony Brook, NY 11794-2100, USA; 3 Department of Biological Sciences, Stony Brook University, Stony Brook, NY 11794, USA; 4 Department of Pathology, Stony Brook University Hospital, Stony Brook, NY 11794, USA; 5 National Research Centre for the Working Environment, Lerso Parkalle 105, 2100 Copenhagen, Denmark

## Abstract

**Background:**

Given the importance of highly reactive oxygen species (hROS) as reactants in a wide range of biological, photochemical, and environmental systems there is an interest in detection and quantification of these species. The extreme reactivity of the hROS, which includes hydroxyl radicals, presents an analytical challenge. 3'-(*p*-Aminophenyl) fluorescein (APF) is a relatively new probe used for measuring hROS. Here, we further evaluate the use of APF as a method for the detection of hydroxyl radicals in particle suspensions.

**Results:**

Particle-generated hROS can be quantified with an estimated detection limit of 50 nM. Measurements of hROS in two National Institute of Standards and Technology (NIST 2709 and 2710) soil suspensions and a pyrite suspension show non-linear particle dose-response curves for hROS generation. APF can also be used in solutions containing no dissolved molecular oxygen (O_2_) to determine the role of O_2 _in the formation of hROS. Results confirm that O_2 _is mechanistically important in the formation of hROS by dissolved ferrous iron and in pyrite suspensions.

**Conclusion:**

Given the non-linear dose-response curves for particle generation of hROS, we recommend using several particle loadings in experiments aimed to compare particles for their hROS generation potential. The method presented here is specific to hROS and simple to perform. The analysis can be conducted in mobile labs as only basic laboratory equipment is required.

## Background

Hydroxyl radicals are a highly reactive oxygen species (hROS) that reacts non-specifically with most organic molecules within nanoseconds after their formation [1]. *In vivo*, hydroxyl radicals (•OH) have been implicated in causing oxidative stress [2,3] and several diseases [4,5]. The role of particulate-induced formation of •OH on inducing lung diseases has been the focus of many studies. Hydroxyl radicals have been directly implicated in lung diseases related to exposures to asbestos [6], silica [7,8], and other airborne particulate matter [9,10] as a result of genotoxicity [7,2] and/or oxidative stress [11,2,12]. Hence, ^•^OH formation *in vitro *and *in vivo *has been used as an indicator for particulate-induced toxicity potential [7,2,11,13,4,15].

The mechanisms whereby particles induce the formation of hROS in solution and *in vivo *are not fully understood, however several pathways are recognized [for a review, see [16]]. In solution, particles containing transition metals may generate •OH by redox reactions involving metals exposed at the particle surface or by redox reactions with metal ions released from the particles to solution. The reaction of ferrous iron with dissolved molecular oxygen serves as an example. Ferrous iron reacts with molecular oxygen to form hydrogen peroxide (eqs 1 and 2). This hydrogen peroxide can then react with ferrous iron to form hydroxyl radicals through the Fenton reaction (eq. 3).(1)(2)(3)

Peroxy radicals can be formed from reactions involving hydrogen peroxide and iron or hydroxyl radicals (eqs 4 and 5).(4)(5)

These reaction sequences are not the only mechanisms by which particulates can lead to hROS formation. Mineral defects may also contribute to the formation of hydroxyl radicals in solution. In the process of crushing quarts and other silicates, the bonds separating atoms cleave homolytically resulting in single electrons at each atom. These lone electrons react with molecular oxygen or water to form hydroxyl radicals [17,18]. Inhaled particles can generate •OH via the previously mentioned mechanisms in addition to cellular-mediated processes. As an immune system defense against invading bacteria, certain cells (e.g., macrophages) will engulf (i.e., phagocytosis) bacteria and destroy them by exposure to lysozymes and ROS. However, the lysozymes and ROS will have little effect on a particle, resulting in a continued immune response and formation of ROS including •OH (for reviews see [11,13,19,3]).

The extremely short half-life of hROS presents an analytical challenge to their quantification and precludes direct detection [1]. With direct detection precluded, methods have been developed that rely on analyzing the product of a reaction between hROS and a target molecule. In some techniques, the target molecule will oxidize and change color (e.g., leuco crystal violet) [20] or other target molecules will fluoresce when oxidized by hROS [e.g., 2',7'-dichlorofluorescein (DCFH)] [21-23]. In other methods using electron spin resonance (ESR), the hydroxyl radical will be "trapped" (i.e., added to) on a larger molecule, the "spin trap" [e.g., 5,5-dimethyl, 1-pyrroline *N*-oxide (DMPO)], which becomes a relatively more stable radical species (i.e., DMPO-OH) that can be detected [24-27]. In the presence of cells or in tissue, the products of particle-induced radical oxidation include lipid peroxidation [28], DNA strand-breaks [29,7], RNA degradation [30], nucleobase oxidation [31,31,33] and upregulation of signaling molecules indicative of inflammation and apoptosis (i.e., programmed cell death) such as cytokines and p53 [34-36].

Although several techniques are available for the detection of hROS in solution, none of the techniques are suitable for all experiments; some techniques are better adapted to cellular systems and others to cell-free solutions. Spin-trapping using ESR and the spin-trap DMPO has been used extensively but it requires specialized equipment (i.e., an ESR spectrometer) and the method is susceptible to artifacts when ferric iron is present [e.g., Fe(III) associated with a particle] [37]. In addition, DMPO-OH has a short half-life (i.e., 61.2 minutes [38] without cells and 2.9 minutes in the presence of cells [39]). Other techniques for hROS detection involve the hROS-induced oxidation of a molecule, which results in a change to the target molecule's fluorescence. The most commonly used fluorogenic probe is 2',7'-dichlorofluorescein (DCFH) which will react with several reactive oxygen species (ROS), not just hROS [23]. Since DCFH will not react with reactive oxygen species unless it undergoes a de-esterification step either within cells or chemically [22], its use with particle suspensions in the absence of cells requires an extra de-esterification step. Sodium terephthalate has also been used as a fluorescence probe for assessing the Fenton reaction in extracellular fluid [40] and *in vivo *using HPLC [41]. Assessing the fate of nucleic acids after exposure to particles is another technique that has been employed for the detection of particle-induced formation of hROS [29,7,30]. Although this method is useful for determining the genotoxic potential of particles, it is not well adapted for quantifying the hROS concentration that is generated. In addition, when cellular DNA degradation is evaluated, DNA strand repair mechanisms will reduce the level of particle ROS-induced strand breaks [42]. Compared to the current methods for hROS detection, a relatively new compound and detection technique shows promise for greater specificity, simplified experimental protocols, and requirement for standard laboratory equipment (i.e., fluorometer).

3'-(*p*-Aminophenyl) fluorescein (APF) is a non-fluorescent molecule until it is reacted with either hydroxyl radicals, hypochlorite (^-^OCl), peroxynitrite anions (ONOO^-^) [21] or peroxy radicals [43]. APF will also be transformed into the fluorescent form if exposed to a combination of H_2_O_2 _and horseradish peroxidase (HRP); HRP catalyzes the oxidation of APF by H_2_O_2_. In a solution containing both H_2_O_2 _and ^•^OH, only ^•^OH will react with the APF unless HRP is added. APF has been used for detecting tobacco-induced intracellular peroxynitrite [44] and we have successfully used APF for detecting ^•^OH generated in mineral slurries [45] and coal aqueous suspensions [46]. The objectives of this contribution are: 1) to further examine the usefulness of using APF in the presence of particulates and in solution compositions that may lead to the formation of hROS (e.g., containing ferrous iron), 2) evaluating the kinetics of its reactivity, 3) the pH-dependence of the APF sensitivity, and 4) the effect of particle loading on hROS generation.

In order to evaluate the usefulness of APF as a detection tool for solution and particulate-induced formation of hROS, several experiments were performed. The sensitivity of APF to reactions with Fenton generated OH was determined as well as the HRP-catalyzed oxidation of APF by H_2_O_2_. Ferrous iron induced formation of OH (eqs 1 to 3) was also evaluated along with the kinetics of these reactions and pH-dependence. To evaluate the use of the APF technique for detecting particulate-induced OH, experiments were performed as a function of particle loading. These experiments were conducted with pyrite, a mineral that has been previously shown to form OH [47,30,46], and two National Institute of Standards and Technology (NIST) soil standards that have an unknown potential for hROS generation.

## Results

**Figure 1:**
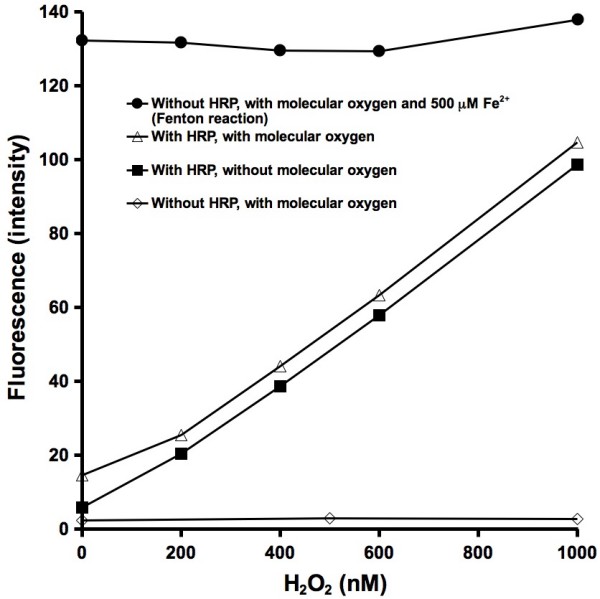
**Reaction of 10 μM aminophenyl fluorescein (APF), 50 mM phosphate pH 7.40 buffer, H_2_O_2_, and 0.2 μM horseradish peroxidase (HRP)**. The experiment was performed aerobically and in the absence of molecular oxygen. The top curve (HRP and molecular oxygen) is used as a calibration curve for all further experiments and expressed as "hROS reactivity (nM H_2_O_2_)". Fluorescence expressed as arbitrary units.

APF is oxidized by H_2_O_2 _in the presence of HRP. Increasing the concentration of H_2_O_2 _results in an increase in APF fluorescence (Figure 1). When the reactant solutions (i.e., water, APF, pH buffer, HRP, H_2_O_2_) are purged with nitrogen gas and the experiment is performed in an anaerobic glove-bag, a similar fluorescence increase results but at a slight decrease in overall fluorescence. Without HRP, the fluorescence does not increase with addition of H_2_O_2_.

**Figure 2:**
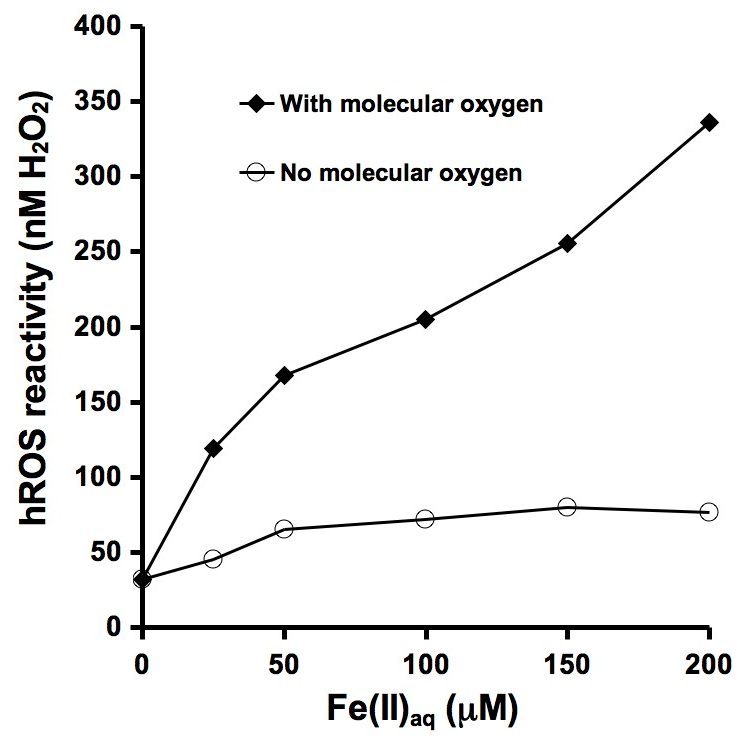
**Ferrous iron-induced generation of hydroxyl radicals in the presence of 10 μM APF and 50 mM phosphate pH 7.40 buffer through the Fe/O_2 _and Fenton reactions at room temperature (22 ± 2°C)**. Ferrous iron was added as a sulfate salt. The experiment was repeated in the absence of molecular oxygen. The hROS reactivity values can be compared to the reported fluorescence intensity values shown on the other figures by dividing hROS reactivity by ten.

In solution, ferrous iron reacts with molecular oxygen to form hydroxyl radicals (eqs 1 to 3). When increasing amounts of ferrous iron are added to a solution containing APF, the concentration of hydroxyl radicals increases (Figure 2). When the experiment is repeated in the absence of molecular oxygen, the same increase in OH formation is not observed.

**Figure 3:**
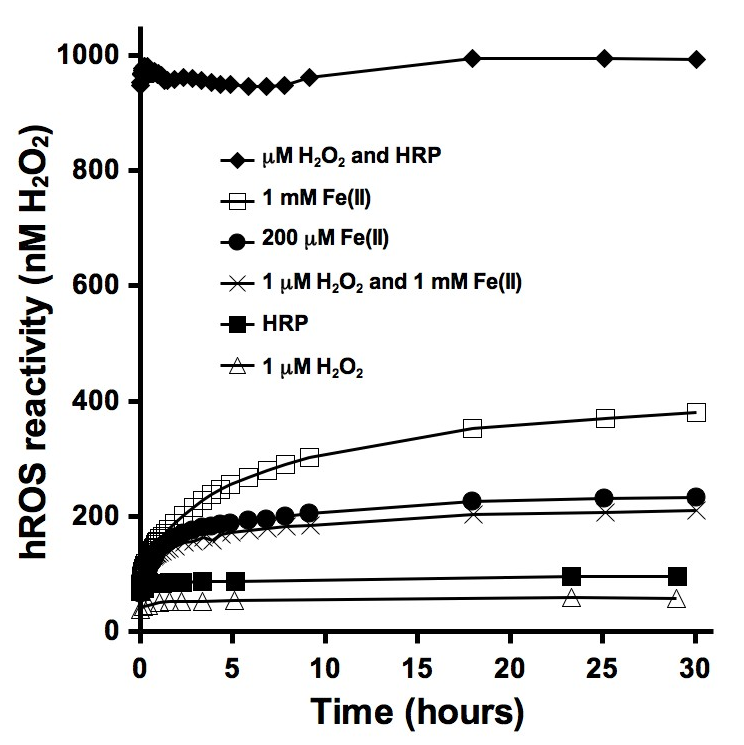
**Kinetic analysis of several reactants in the presence of 10 μM APF and 50 mM phosphate pH 7.40 buffer at room temperature (22 ± 2°C)**. Fluorescence measurements were taken as a function of time. The hROS reactivity values can be compared to the reported fluorescence intensity values shown on the other figures by dividing hROS reactivity by ten.

The formation of hydroxyl radical was determined under a range of conditions as a function of incubation time (Figure 3). All of the experiments were performed in the presence of APF and pH buffer. When HRP is added to a 1000 nM H_2_O_2 _solution, APF is oxidized within minutes resulting in a fluorescence intensity that approaches the corresponding calibration value of 1000 nM hROS (i.e., within 1 minute after adding reactants, the fluorescence comes to within 4.5% of the value that it will in 24 hours). After 24 hours, the fluorescence intensity translates to an hROS concentration of 994 nM, which is a 0.6% error from the calibrated value of 1000. Reaction of 1 mM and 200 μM ferrous iron show similar increases in hROS generation as a function of time. Compared to the preceding reaction with HRP, this reaction is slower. When both H_2_O_2 _and ferrous iron are added, the resulting concentration of hROS does not reach the expected level of at least 1000 nM hROS. When either HRP or H_2_O_2 _are added alone, the fluorescence intensity and values expressed as hROS concentration are low.

**Figure 4:**
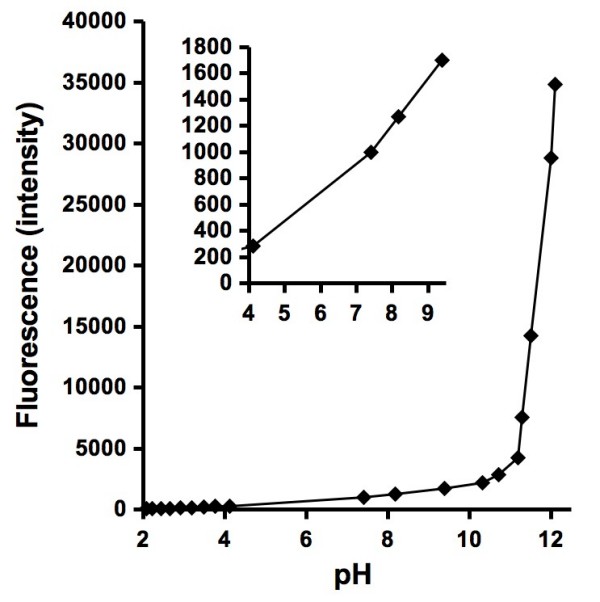
**Effect of pH on the fluorescence intensity of 1 μM H_2_O_2 _reacting with 10 μM APF in the presence of 0.2 μM HRP and addition of either hydrochloric acid or sodium hydroxide for 24 hours**. Inset shows the fluorescence intensity from pH 4 to 9. The fluorescence intensities roughly correspond to nM of H_2_O_2 _that would react with HRP, so that a fluorescence of 1000 equates to 1000 nM H_2_O_2_.

In unbuffered and weakly-buffered solutions, the pH will affect the fluorescence intensity of APF. This may be of particular concern when certain particles or high loadings of particles are used in experiments. To determine how pH affects the fluorescence intensity, 1 μM H_2_O_2_, APF, and HRP were incubated with either addition of hydrochloric acid or sodium hydroxide. After 24 hours, the fluorescence was measured, followed by pH measurements. The results show that as the pH is increased, so does the fluorescence intensity (Figure 4). The effect is significant when the pH is raised above 11.

**Figure 5:**
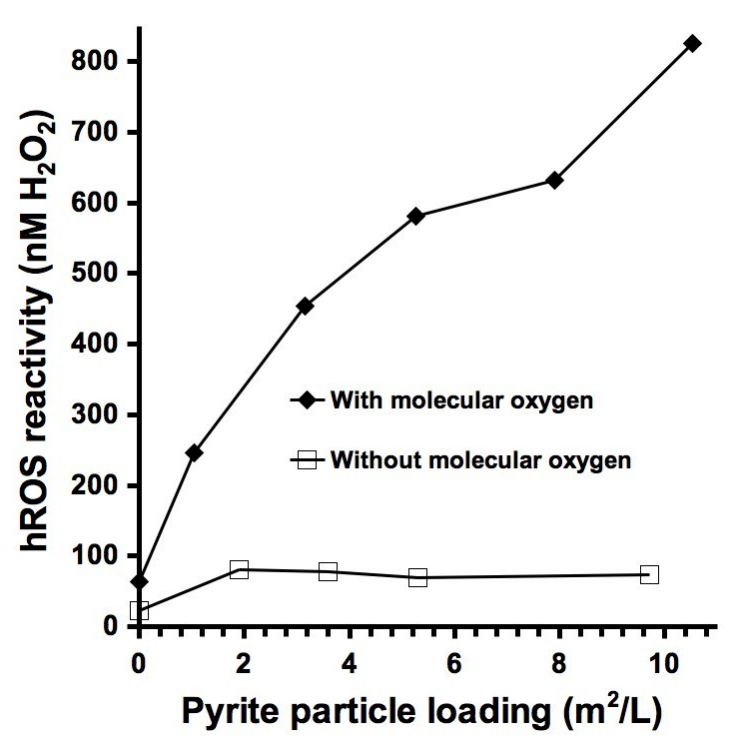
**Pyrite generated hydroxyl radicals as a function of pyrite surface area loading**. Pyrite particles (roughly 63-38 microns in diameter) were incubated with 10 μM APF and 50 mM phosphate pH 7.40 buffer for 24 hours followed by filtration (0.45 μm) and fluorescence measurements. The hROS reactivity values can be compared to the reported fluorescence intensity values shown on the other figures by dividing hROS reactivity by ten.

Pyrite has been previously shown to form hydroxyl radicals when it is in aqueous suspension using ESR spin-trapping with DMPO [30]. To evaluate the effect of pyrite surface area on hROS formation, several different loadings of crushed pyrite were incubated with APF and pH buffer. After 24 hours and filtration, higher loadings of pyrite resulted in larger concentrations of detected hROS (Figure 5). The mechanism of pyrite-generated hROS is still not completely understood, however the reaction of ferrous iron either dissolved from pyrite or at its surface with molecular oxygen is a plausible mechanism. The experiment with pyrite was repeated in the absence of molecular oxygen to test this hypothesis. The results show that the hROS concentration is low and does not increase with increasing loadings of pyrite suggesting that either hROS is not formed or it is formed at very low concentrations.

**Figure 6:**
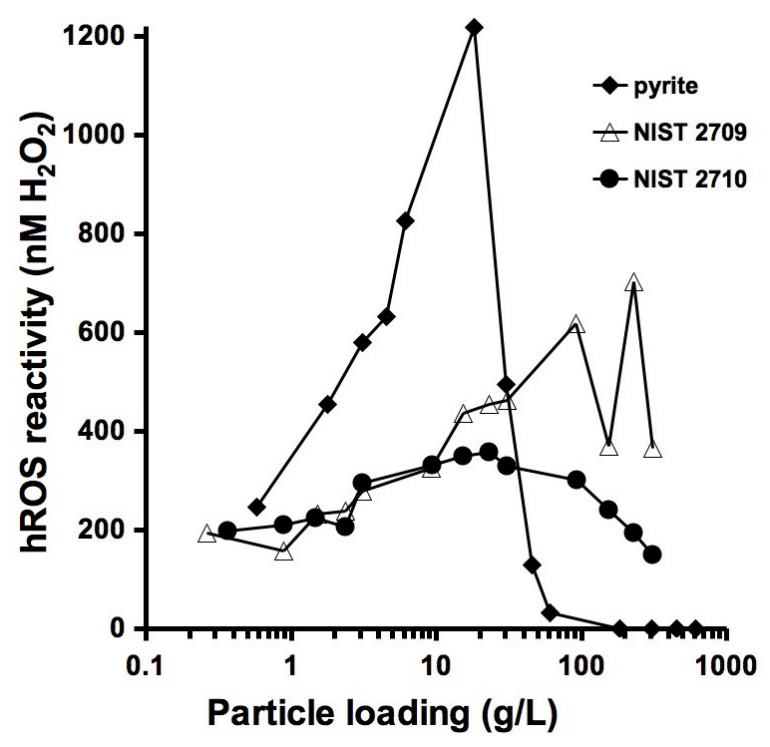
**Hydroxyl radical generation as a function of particle loadings**. Mineral particles (roughly 1–100 microns in diameter) were incubated with 10 μM APF and 50 mM phosphate pH 7.40 buffer for 24 hours followed by filtration (0.45 μm) and fluorescence measurements. The National Institute of Standards and Technology (NIST) samples used here are a low metal-content soil, San Joaquin soil (2709), and a high metal-content soil, Montana 1 (2710). The hROS reactivity values can be compared to the reported fluorescence intensity values shown on the other figures by dividing hROS reactivity by ten.

In order to determine how particle loadings affect the concentration of hROS detected, a range of loadings of pyrite and two NIST soil standards were incubated with APF and pH buffer (Figure 6). The concentration of hROS generated by pyrite increases until the particle loading reaches around 13 g/L. Increasing the loading beyond 13 g/L results in a decrease in the APF fluorescence. Similar trends were observed with both of the NIST standards.

## Discussion

One of the main reasons for evaluating the use of APF for detection of hROS is its potential for semi-quantifying concentrations of particulate-generated OH. Reacting known concentrations of OH with APF would provide the most direct and accurate calibration curve but generating solutions with precise concentrations of OH is not easy. Fenton generated OH is based on the reaction of ferrous iron with H_2_O_2 _(eq. 3). The Fenton reaction cannot be used as a basis for calibration of the APF technique because the presence of molecular oxygen in a ferrous iron solution will lead to the formation of additional hydrogen peroxide (eqs 1 and 2). These side reactions could lead to a higher concentration of OH than expected on the basis of the added hydrogen peroxide. Results presented here suggest that the formation of additional OH is not a significant factor, in fact, the opposite is observed: the APF response is lower than expected. It is not clear what causes this, but the fluorescence generated by a 1000 nM H_2_O_2 _and 1 mM ferrous iron solution is less than expected (Figure 3). Either the Fenton reaction is not complete or dissolved ferrous iron is reacting with OH (eq. 6).(6)

For these reasons, the calibration curve that was used is based on the enzymatic reaction of H_2_O_2 _and HRP. Although the reaction between H_2_O_2 _and HRP does not produce a known amount of OH, it does produce a repeatable reactivity that will oxidize APF in a H_2_O_2_-dose dependent manner.

The results from experiments with ferrous iron (Figure 2) and pyrite (Figure 5) show similar results. The concentration of hROS that is formed increases with the concentration of ferrous iron and pyrite loadings and the OH-generating reactions are both dependent on molecular oxygen. This suggests that the OH-generating mechanisms are based on the Fe/O_2 _and Fenton reactions (eqs 1 to 3). The anoxic experiment with pyrite shows a particle loading-independent detection of hROS. The formation of hROS in the absence of molecular oxygen may be due to reaction of pyrite-associated ferrous iron with residual H_2_O_2 _in the water used in the experiments, which can form OH (eq. 3).

The rates of APF conversion vary considerably (Figure 3). The enzymatic oxidation reaction between H_2_O_2 _and HRP is complete within minutes. As shown in Figure 3, there is no significant difference in fluorescence between the first measurement taken within minutes of the start of the reaction and a measurement taken after 30 hours. By contrast all other experiments show a rise in fluorescence over time. The experiments with dissolved ferrous iron show a dependence on the iron concentration. The oxidation of APF is cumulative over time, therefore these curves show that more hROS are being formed in the first few hours of the reaction and that smaller amounts of hROS are formed as the reaction proceeds. A comparison between the resulting curves generated by addition of either HRP or H_2_O_2 _to a blank solution shows that addition of HRP results in slightly more hROS. A small amount of H_2_O_2 _may be present in the water that was used in the experiments. APF is not expected to react with H_2_O_2 _[21]. APF does have a slight fluorescence at the time when it is purchased. Due to the variations in APF fluorescence and the presence of low concentrations of H_2_O_2 _in water the technique has an estimated lower detection limit around 50 nM hROS.

The fluorescence intensity from APF that has been oxidized by H_2_O_2 _is pH dependent (Figure 4). With the addition of a pH buffer, this should not present a problem, however some chemical reactants or reactive particulates will affect the pH. Therefore, we recommend that the pH be measured following all experiments. The cause for the pH-dependent increase in fluorescence from the experiment shown in Figure 4 remains unknown. The increase in fluorescence may be due to changes in the fluorescence properties of APF or HRP, or an increased reactivity between APF, HRP and H_2_O_2_. As the pH in solutions containing APF is increased from acidic values to 10, the fluorescence gradually increases. Below a pH around 4 or 5, the fluorescence is weak suggesting that it is best to use APF in solutions with pH values between 5 and 10.

To gain insight into the mechanism that results in the formation of hROS within pyrite suspensions, experiments were performed with the presence and absence of molecular oxygen (O_2_) (Figure 5). Dissolved molecular oxygen is a reactant in the Fe/O_2 _reaction that produces hydrogen peroxide (eqs 1 and 2). By repeating an experiment with pyrite in the absence of O_2_, the role of the Fe/O_2 _reaction in generating OH within pyrite suspensions was evaluated. As the pyrite surface area (and also particle loading in g/L) is increased in the presence of O_2_, the observed hROS concentrations also increase. However, in the absence of O_2_, the same increase in hROS does not occur. This indicates that O_2 _is important in the mechanism that leads to the formation of OH and other hROS.

Particle loadings have a significant effect on the concentration of detected hROS. The loading of particles that are used in experiments are often based on a relevant environmental loading (e.g., lung exposure) or a loading that is suitable to the experiment (i.e., a particulate amount that can be accurately weighed or a loading that will result in a detectable fluorescence). As shown in Figure 5, as the pyrite loading is increased in the presence of O_2_, the concentration of observed hROS also increases. However, a particle loading-dependent increase in observed hROS does not always occur. For example, in Figure 6, pyrite at 100 m^2^/L shows no generation of hROS while at a loading of 53 m^2^/L, 495 nM of hROS is formed. Without knowing an appropriate particulate loading and performing an experiment with only one loading, the resulting concentration of hROS that is formed may not be representative for this particulate at other loadings. Therefore, we recommend performing experiments with several particle loadings. A decrease in observed hROS with increasing pyrite particle loadings (i.e., above 20 g/L) may be due to several factors including adsorption of APF to the higher particle surface area, alteration of the solution pH, or further APF oxidation by an overwhelming concentration of hROS. The decrease in observed hROS with a pyrite loading above 20 g/L appears to occur suddenly, but the pyrite loading is shown on a logarithmic-scale (i.e., the decrease in hROS may appear more gradual on a linear scale). The mechanisms that lead to the formation of hROS by the NIST soil standards may be due to the presence of ferrous iron, however the specific mechanism remains unresolved.

## Conclusion

Measurement of hROS in particle suspensions can be achieved by observing the fluorescence of a probe molecule, APF, that is incubated with the particle suspension. The APF technique selectively detects only highly-reactive ROS (e.g., hydroxyl radicals, peroxy radicals, peroxynirite anions, and hypochlorite anions) and it has an estimated detection limit is of 50 nM. The specificity of APF for hydroxyl radicals and other hROS could be useful for evaluating the mechanisms of ROS generation and determining which particular ROS species is generated in a particle suspension. Compared to our previous studies using other fluorescence [42] and ESR spin-trapping [30] techniques for quantifying the particle-induced formation of hROS, the APF method has a low detection limit, does not require specialized equipment, and APF can be used in particle suspensions incubated for minutes to more than a day.

### Experimental

#### Highly-reactive oxygen species measurements

3'-(*p*-aminophenyl) fluorescein (APF) from Invitrogen™ was used for the detection of hROS. A calibration curve was generated by incubating a known amount of H_2_O_2 _with 10 μM APF, 50 mM potassium phosphate buffer at pH 7.40, and 2.95 units/mL (equivalent to 0.2 μM) Sigma type II horseradish peroxidase (HRP) in a 2-mL centrifuge tube. The tubes were closed and placed on an end-over-end rotator in the dark at room temperature (22 ± 2°C). After 24 hours, the solutions were transferred to 4-mL methylcrylate fluorescence cuvettes followed by fluorescence measurements using a Tuner Barnstead spectrofluorometer with excitation and emission wavelengths set to 490 nm and 520 nm, respectively. Using the calibration curve generated in this way, the fluorescence data from other experiments is presented on the figures as "hROS reactivity (nM H_2_O_2_)". In experiments designed to determine hROS generation from minerals, powdered minerals were incubated in 2-mL centrifuge tubes and rotated end-over-end with water (Easy Pure 18.3 MΩ-cm, UV-irradiated, ultrafiltered),10 μM APF, and 50 mM potassium phosphate buffer at pH 7.40 at room temperature (22 ± 2°C). After 24 hours, the suspensions were filtered (Millipore PVDF 0.45 μm) followed by fluorescence measurements. For experiments that were performed in the absence of molecular oxygen, all solutions were purged with nitrogen gas for 30 minutes to remove molecular oxygen. The solutions were transferred into a glove bag with an atmosphere of 97% nitrogen and 3% hydrogen with palladium catalyst (Coy Laboratory Products) to remove gaseous molecular oxygen. The various reactants were incubated in 2-mL centrifuge tubes and rotated end-over-end in the dark and transferred to cuvettes after 24 hours. The tops of the cuvettes were covered with a flexible film (Parafilm M) before taking them out of the anaerobic glove-bag followed by immediate fluorescence measurements.

#### Mineral sample preparation and soil standards

Natural pyrite (Huanzala, Peru) obtained from Wards was crushed in an agate mill. After crushing it was sieved between 38 to 63 μm and stored in a vacuum dessicator until used in the experiments. In earlier work with this same size fraction we determined a specific surface area of 1.25 m^2^/g using a five-point N_2 _adsorption BET. Soil standards # 2709 and 2710 were purchased from the National Institutes of Standards and Technology (NIST) and used without any treatment. NIST 2709 is a soil with baseline trace element concentrations from a plowed field in the central Californian Joaquin valley. NIST 2710 is a soil from Montana with highly elevated trace element concentrations. The sample was gathered from pastureland that was periodically flooded by a nearby creek with high concentrations of copper, manganese, and zinc. Further preparation and characterization details on the NIST standards are located on the NIST website: http://www.nist.gov/srm. 

## Abbreviations

(DCFH): 2',7'-dichlorofluorescein; (APF): 3'-(*p*-Aminophenyl) fluorescein; (^•^OH): hydroxyl radicals; (^-^OCl): hypochlorite; (ONOO^-^): peroxynitrite anions; (HRP): horseradish peroxidase; (DMPO): 5,5-dimethyl, 1-pyrroline *N*-oxide; (hROS): highly reactive oxygen species; (ESR): electron spin resonance; (NIST): National Institute of Standards and Technology.

## Competing interests

The authors declare that they have no competing interests.

## Authors' contributions

CAC helped design the study, perform most experiments, and drafted the manuscript, CEP performed many experiments, SNH performed some experiments, SRS helped design the study, and MAAS provided funding, helped design and supervise the study. All authors have read and approved the final manuscript.

## References

[B1] Pryor WA Oxy-radicals and related species: their formation, lifetimes, and reactions Annual Review of Physiology 1986 48 657 663 10.1146/annurev.ph.48.030186.003301 3010829

[B2] Donaldson K Stone V Borm PJA Jimenez LA Gilmour PS Schins RPF Knaapen AM Rahman I Faux SP Brown DM MacNee W Oxidative stress and calcium signaling in the adverse effects of environmental particles (PM10) Free Radical Biology and Medicine 2003 34 1369 1382 10.1016/S0891-5849(03)00150-3 12757847

[B3] Babior BM Phagocytes and oxidative stress American Journal of Medicine 2000 109 33 44 10.1016/S0002-9343(00)00481-2 10936476

[B4] Halliwell B Role of free radicals in the neurodegenerative diseases – Therapeutic implications for antioxidant treatment Drugs & Aging 2001 18 685 716 11599635 10.2165/00002512-200118090-00004

[B5] Halliwell B Gutteridge JMC Role of free radicals and catalytic metal ions in human disease: an overview Methods in Enzymology 1990 189 1 85 10.1016/0076-6879(90)86093-b 2172697

[B6] Kamp DW Graceffa P Pryor WA Weitzman SA The role of free radicals in asbestos-induced diseases Free Radical Biology and Medicine 1992 12 293 315 1577332 10.1016/0891-5849(92)90117-y

[B7] Vallyathan V Shi X Castranova V Reactive oxygen species: Their relation to pneumoconiosis and carcinogenesis Environmental Health Perspectives 1998 106 1151 1156 9788890 10.1289/ehp.98106s51151 PMC1533374

[B8] Fubini B Zanetti G Altilia S Tiozzo R Lison D Saffiotti U Relationship between surface properties and cellular responses to crystalline silica: studies with heat-treated cristobalite Chem Res Toxicol 1999 12 737 745 10458708 10.1021/tx980261a

[B9] Knaapen AM Borm PJA Albrecht C Schins PF Inhaled particles and lung cancer. Part A: mechanisms International Journal of Cancer 2004 109 799 809 10.1002/ijc.11708 15027112

[B10] Dellinger B Pryor WA Cueto R Squadrito GL Hegde V Deutsch WA Role of free radicals in the toxicity of airborne fine particulate matter Chem Res Toxicol 2001 14 1371 1377 11599928 10.1021/tx010050x

[B11] Fubini B Hubbard A Reactive oxygen species (ROS) and reactive nitrogen species (RNS) generation by silica in inflammation and fibrosis Free Radical Biology and Medicine 2003 34 1507 1516 12788471 10.1016/s0891-5849(03)00149-7

[B12] Risom L Moller P Loft S Oxidative stress-induced DNA damage by particulate air pollution Mutation Research 2005 592 119 137 16085126 10.1016/j.mrfmmm.2005.06.012

[B13] Kamp DW Graceffa P Pryor WA Weitzman SA The Role of Free-Radicals in Asbestos-Induced Diseases Free Radical Biology and Medicine 1992 12 293 315 1577332 10.1016/0891-5849(92)90117-y

[B14] Halliwell B Aruoma OI DNA damage by oxygen-derived species FEBS Letters 1991 281 9 19 1849843 10.1016/0014-5793(91)80347-6

[B15] Donaldson K Brown DM Mitchell C Dineva M Beswick PH Gilmour P MacNee W Free radical activity of PM10: Iron-mediated generation of hydroxyl radicals Environ Health Perspect 1997 105 1285 1289 9400739 10.1289/ehp.97105s51285 PMC1470141

[B16] Schoonen MAA Cohn CA Roemer E Laffers R Simon S O'Riordan T Mineral-induced formation of reactive oxygen species Reviews in Mineralogy and Geochemistry 2006 64 179 221

[B17] Fubini B Bolis V Giamello E The surface chemistry of crushed quartz dust in relation to its pathogenicity Inorganica Chimica Acta 1987 138 193 197

[B18] Hasegawa M Ogata T Sato M Mechano-radicals produced from ground quartz and quartz glass Powder Technology 1995 85 269 274

[B19] Djordjevic VB Free radicals in cell biology International Review of Cytology – a Survey of Cell Biology 2004 237 57 89 10.1016/S0074-7696(04)37002-6 15380666

[B20] Cohn CA Pak A Schoonen MAA Strongin DR Quantifying hydrogen peroxide in iron-containing solutions using leuco crystal violet Geochemical Transactions 2005 6 47 52 10.1186/1467-4866-6-47 PMC1475790 35412761

[B21] Setsukinai K Urano Y Kakinuma K Majima HJ Nagano T Development of novel fluorescence probes that can reliably detect reactive oxygen species and distinguish different species J Biol Chem 2003 278 3170 3175 12419811 10.1074/jbc.M209264200

[B22] LeBel CP Ischiropoulos H Bondy SC Evaluation of the probe 2',7'-dichlorofluorescin as an indicator of reactive oxygen species formation and oxidative stress Chemical Research in Toxicology 1992 5 227 231 1322737 10.1021/tx00026a012

[B23] Rota C Chignell CF Mason RP Evidence for free radical formation during the oxidation of 2'-7'-dichlorofluorescin to the fluorescent dye 2'-7'-dichlorofluorescein by horseradish peroxidase: possible implications for oxidative stress measurements Free Radical Biology & Medicine 1999 27 873 881 10515592 10.1016/s0891-5849(99)00137-9

[B24] Fubini B Mollo L Giamello E Free radical generation at the solid/liquid interface in iron containing minerals Free Radical Research 1995 23 593 614 8574353 10.3109/10715769509065280

[B25] Shi H Hudson LG Ding W Wang S Cooper KL Liu S Chen Y Shi X Liu KJ Arsenite causes DNA damage in keratinocytes via generation of hydroxyl radicals Chem Res Toxicol 2004 17 871 878 15257611 10.1021/tx049939e

[B26] Borisenko GG Martin I Zhao Q Amoscato AA Tyrunia YY Kagan VE Glutathione propagates oxidative stress triggered by myeloperoxidase in HL-60 cells J Biol Chem 2004 279 23453 23462 15039448 10.1074/jbc.M400119200

[B27] Blough NV Simpson DJ Chemically mediated fluorescence yield switching in nitroxide-fluorophore adducts: optical sensors of radical/redox reactions J Am Chem Soc 1988 110 1915 1917

[B28] Vallyathan V Generation of oxygen radicals by minerals and its correlation to cytotoxicity Environmental Health Perspectives Supplements 1994 102 1 9 10.1289/ehp.94102s10111 PMC1566993 7705284

[B29] Schins R Mechanisms of genotoxicity of particles and fibers Inhal Toxicol 2002 14 57 78 12122560 10.1080/089583701753338631

[B30] Cohn C Mueller S Wimmer E Leifer N Greenbaum S Strongin DR Schoonen M Pyrite-induced hydroxyl radical formation and its effect on nucleic acids Geochemical Transactions 2006 7 3 16759350 10.1186/1467-4866-7-3 PMC1523326

[B31] Sorensen M Autrup H Hertel O Wallin H Knudsen LE Loft S Personal exposure to PM2.5 and biomarkers of DNA damage Cancer Epidemiology Biomarkers & Prevention 2003 12 191 196 12646506

[B32] Knaapen AM Seiler F Schilderman P Nehls P Bruch J Schins RPF Borm PJA Neutrophils cause oxidative DNA damage in alveolar epithelial cells Free Radical Biology and Medicine 1999 27 234 240 10443941 10.1016/s0891-5849(98)00285-8

[B33] Karlsson HL Nilsson L Moller L Subway particles are more genotoxic than street particles and induce oxidative stress in cultured human lung cells Chemical Research in Toxicology 2005 18 19 23 15651844 10.1021/tx049723c

[B34] Hetland RB Cassee FR Refsnes M Schwarze PE Lag M Boere AJF Dybing E Release of inflammatory cytokines, cell toxicity and apoptosis in epithelial lung cells after exposure to ambient air particles of different size fractions Toxicology in Vitro 2004 18 203 212 14757111 10.1016/s0887-2333(03)00142-5

[B35] Driscoll KE Carter JM Hassenbein DG Howard B Cytokines and particle-induced inflammatory cell recruitment Environ Health Perspect 1997 105 Suppl 5 1159 1164 9400717 10.1289/ehp.97105s51159 PMC1470151

[B36] Panduri V Surapureddi S Soberanes S Weitzman SA Chandel N Kamp DW P53 mediates amosite asbestos-induced alveolar epithelial cell mitochondria-regulated apoptosis Am J Respir Cell Mol Biol 2006 34 443 452 16357363 10.1165/rcmb.2005-0352OC PMC2644206

[B37] Makino K Hagiwara T Hagi A Nishi M Murakami A Cautionary note for DMPO spin trapping in the presence of iron ion Biochemical and Biophysical Research Communications 1990 172 1073 1080 2173913 10.1016/0006-291x(90)91556-8

[B38] Tsai P Pou S Straus R Rosen GM Evaluation of various spin traps for the in vivo in situ detection of hydroxyl radical Journal of the Chemical Society, Perkin Transactions 2 1999 2 1759 1763

[B39] Khan N Wilmot CM Rosen GM Demidenko E Sun J Joseph J Hara J Kalyanaraman B Swartz HM Spin traps: in vitro toxicity and stability of radical adducts Free Radical Biology and Medicine 2003 34 1473 1481 12757857 10.1016/s0891-5849(03)00182-5

[B40] Freinbichler W Tipton KF Corte LD Linert W Mechanistic aspects of the Fenton reaction under conditions approximated to the extracellular fluid Journal of Inorganic Biochemistry 2009 103 28 34 18848726 10.1016/j.jinorgbio.2008.08.014

[B41] Freinbichler W Colivicchi MA Fattori M Ballini C Tipton KF Linert W Corte LD Validation of a robust and sensitive method for detecting hydroxyl radical formation together with evoked neurotransmitter release in brain microdialysis Journal of Neurochemistry 2008 105 738 749 18194218 10.1111/j.1471-4159.2007.05168.x

[B42] Collins AR Assays for oxidative stress and antioxidant status: applications to research into the biological effectiveness of polyphenols Am J Clin Nutr 2005 81 261S 267S 15640489 10.1093/ajcn/81.1.261S

[B43] Heyne B Maurel V Scaiano JC Mechanisms of action of sensors for reactive oxygen species based on fluorescein-phenol coupling: the case of 2-[6-4'-hydroxy)phenoxy-3H-xanthen-3-on-9-yl]benzoic acid Organic & Biomolecular Chemistry 2006 4 802 807 16493462 10.1039/b515751j

[B44] Saito S Yamamoto-Katou A Yoshioka H Doke N Kawakita K Peroxynitrite generation and tyrosine nitration in defense responses in tobacco BY-2 cells Plant Cell Physiol 2006 47 689 697 16556649 10.1093/pcp/pcj038

[B45] Cohn CA Simon SR Schoonen MAA Comparison of fluorescence-based techniques for the measurement of hydroxyl radicals in particle suspensions Particle and Fibre Toxicology 2008 5 2 18307787 10.1186/1743-8977-5-2 PMC2289838

[B46] Cohn CA Laffers R Simon S ORiordan T Schoonen MAA Role of pyrite in formation of hydroxyl radicals in coal: possible implications for human health Particle and Fibre Toxicology 2006 3 16 17177987 10.1186/1743-8977-3-16 PMC1764420

[B47] Borda MJ Elsetinow AR Strongin DR Schoonen MA A mechanism for the production of hydroxyl radical at surface defect sites on pyrite Geochimica et Cosmochimica Acta 2003 67 935 939

